# The *mBio* Early-Career Editorial Board Reviewer Training Program: cultivating excellence in peer review

**DOI:** 10.1128/mbio.01763-24

**Published:** 2024-06-14

**Authors:** Arturo Casadevall, Maisha Miles, Miriam Day, Amanda Donaldson

**Affiliations:** 1 Department of Molecular Microbiology and Immunology, Johns Hopkins Bloomberg School of Public Health, Baltimore, Maryland, USA; 2 American Society for Microbiology, Washington, DC, USA

## EDITORIAL

The process of peer review is critical to the proper functioning of science (1), but there are remarkably few opportunities for formal training. In fact, most scientists, including one of your authors (A.C.), learned to review manuscripts when their mentors asked them for help in reviewing or when a journal reached out to them directly (2). This self-taught process is inefficient and uneven and does not benefit from the experience of editors and journals. Reviews done by scientists in training or early-career stages have been described as “frequently full of nit-picky details about the text and word choice with little critical analysis of the scientific content, or they are extremely critical with an indignant tone that can border on hostility” (2). Consequently, journals and scientific organizations have taken an interest in improving peer review by developing resources and courses to train peer reviewers (for example, see https://genetics-gsa.org/career-development/peer-review-training-program/). Interest in improving peer review has led to the development of online courses dedicated to teaching peer review on a broader scale (3). At *mBio*, we went a bit further by creating the Early-Career Editorial Board (ECB), which is supervised by the journal editors and managed by journal staff, to provide hands-on experience in the process of peer review.

### Idea conception

The *mBio* ECB was the brainchild of Maisha Miles, who was the journal’s longtime Managing Editor and later Journal Development Editor. Originally called the Junior Editorial Board (4), we have replaced the word “junior” with “early career” because this more accurately reflects the board’s composition. Maisha conceived the idea of creating a class of early-career scientists who would work with current editors to carefully review manuscripts and receive feedback for their reviews from the editors. The first call for applications went out during the World Microbe Forum in June 2021 and received more than 900 applications, indicating great interest in such a program. In 2023, after deeming the pilot a success, *mBio* launched a call for the second ECB class and received 502 applications from 53 countries (206 U.S. applicants and 296 non-U.S.).

### Promoting diversity and inclusion and creating opportunities for professional growth

In recent years, there has been a movement toward increased inclusivity and diversity in scholarly publishing (5, 6). It is important to *mBio* leadership that the ECB reflects these goals. Of the 64 early-career scientists selected to join the 2022–2023 program, 59% identified as women, and 36% resided outside the United States. Two years later, of the 70 applicants selected to join the 2024–2025 program, half identify as women, and half are from outside the United States, representing 23 countries in total.

Many graduates of the 2022–2023 cohort have since been appointed to the Editorial Boards of other ASM Journals, which illustrates another important purpose of this program: to introduce new researchers to *mBio* and the broader ASM Journals portfolio. By incorporating open calls for applications, the *mBio* ECB program could reach beyond existing contact networks of editors to encourage any interested and qualified individual to apply; therefore, the diversity of its applicant pool increases more than it would have been possible if the process had been guided only by editor nominations. Graduates of the first *mBio* ECB cohort provided the ASM Journals portfolio with a diverse and trained pool of editor candidates who may not have been considered otherwise. Therefore, this program also serves as a pipeline to bring in new and diverse researchers to *mBio* and, more broadly, to ASM.

To better promote the ECB program globally going forward and to share the accomplishments of its members, we launched a new webpage (https://journals.asm.org/journal/mbio/early-career-ed-board) in early 2024. This page includes a comprehensive description of the program, as well as links to several editorials written by ECB members that highlight their experiences (4) and the need to support society journals (7). We encourage everyone to share this page far and wide with their early-career networks, so that we have a diverse pool of applicants when the next call opens in Fall 2025.

### Future of the program

To help shape the future direction of the training program, the outgoing ECB members were surveyed at the end of 2023 on their experience and asked to provide feedback on the program. All but one respondent said they would recommend the ECB to early-career researchers in their networks, and respondents cited increased confidence in reviewing, networking opportunities, and exposure to the editorial process as significant outcomes of the program. They also offered the following valuable ideas for improvement that will be instituted with the second cohort:

- **Mentorship**. The feedback for the educational sessions was overwhelmingly positive, but many indicated they would like more small-group interactions. Therefore, we invited members of the first cohort to serve as mentors to the second cohort to facilitate this. Each mentor has been assigned two or three current ECB members and has agreed to be available to discuss reviews and offer guidance; beyond this, the scope of the mentorship was left for the participants to decide.
- **Consideration of members in different time zones**. We have transitioned some of the live sessions into recorded programming, followed by live Q&A sessions offered at different times to better accommodate all members. Members watch the recorded sessions at their own convenience but still have an opportunity to speak with the presenters at a live Q&A. We encourage members to submit questions beforehand, and we provide summaries afterward for those unable to attend.
- **Adaptable curriculum**. We have added a session about artificial intelligence to the 2024–2025 curriculum, as this is an emerging topic of high interest. We will continue to encourage ECB members to provide feedback throughout the program and will adapt the curriculum to meet their needs.

The traditional role of scientific journals has been to receive scientific reports, have these peer reviewed for rigor and accuracy, and then publish the information to make it freely available. However, times are changing, and journals are increasingly taking additional roles in the scientific enterprise. With the creation of the ECB, *mBio* is following this expanded role for journals by attempting to improve a critical aspect of review and criticism with the hope of enhancing the quality of the scientific literature. As the second ECB class begins its training in peer review and editorial practices, we hope to continue this tradition in the years ahead and perhaps encourage other journals to take similar initiatives.

### Thank you to the inaugural members

The success of this program would not have been possible without the 63 inaugural members who completed the program. They reviewed more than 600 manuscripts during their 2-year tenure, and their feedback has helped shape and improve the second iteration of the program. As mentioned above, many of these dedicated early-career researchers wrote editorials for *mBio*, more than a dozen have joined the Editorial Boards of other ASM journals, and nearly half continue to dedicate their time and experience to mentoring the second class of ECB members. We extend our deepest appreciation to the following members of the inaugural Early-Career Editorial Board:

Cynthia Adinortey

Daniel Aridgides

Sheyda Azimi

Lori Banks

Aaron Barnes

Effie Bastounis

Joseph Boll

Clotilde Bongrand

Germán Bonilla-Rosso

Maria de Fátima Cardoso Pereira

Jiandong Chen

Ousmane Cissé

Valerie Cortez

Sebastien Crepin

Zhouqi Cui

Nsa Dada

Sophie Darch

Nicole De Nisco

Stephen Dolan

Sarah Doore

Elizabeth Draganova

Alison Eastman

Irina El Khoury

Melissa Ellermann

Ryan Gaudet

Alexandre Gouzy

Melinda Grosser

Asiya Gusa

Andrzej Harris

Tania Henriquez

Saheed Imam

Jayhun Lee

Rebeccah Lijek

Rogerio Lourenco

Brian Luna

Bruno Martorelli Di Genova

Renata Matos

Daniel Miller

Angela Mitchell

Roberto Molina-Quiroz

Francisca Nwaokorie

Ceren Ozkul

Katryn Patras

Atmika Paudel

Diana Pires

Diana Proctor

Peter Ramirez

Paulami Rudra

Dan Salamango

Lennart Schada von Borzyskowski

Marie Seraphin

Anna Serquiña

Cecilia Silva-Valenzuela

Om Prakash Singh

Sanhita Sinharay

Liku Tezera

Zulema Udaondo

Rachel Van Duyne

Daniel Vélez-Ramírez

Julia Willett

Jason Yang

Wenqi Yu

Zhengzhong Zou
